# Supplementary material for: Self-establishing communities enable cooperative metabolite exchange in a eukaryote
Source: eLife. 2015 Oct 26;4:e09943. doi: 10.7554/eLife.09943 (PMC4695387; doi:10.7554/eLife.09943)
Supplement: Figure 2—source data 1. — DOI: http://dx.doi.org/10.7554/eLife.09943.007 [file elife-09943-fig2-data1.docx]

Figure 2-source data 1

| **Plasmid** | **Marker** | **Segregation rate (%)** | |
| --- | --- | --- | --- |
|  |  | **Individual** | **All four** |
| p423GPD | *HIS3* | 6.50 ± 0.31 | 3.76 ± 0.30 |
| pRS425 | *LEU2* | 3.81 ± 0.29 | 2.55 ± 0.32 |
| p426GPD | *URA3* | 6.08 ± 0.78 | 2.78 ± 0.67 |
| pRS411 | *MET15* | 1.05 ± 0.25 | 1.89 ± 0.50 |
