# Supplementary material for: Self-establishing communities enable cooperative metabolite exchange in a eukaryote
Source: eLife. 2015 Oct 26;4:e09943. doi: 10.7554/eLife.09943 (PMC4695387; doi:10.7554/eLife.09943)
Supplement: Figure 3—source data 1. — DOI: http://dx.doi.org/10.7554/eLife.09943.013 [file elife-09943-fig3-data1.docx]

Figure 3- source data 1

| **Name** | **Intracellular YSBN5**  **(pmol/ 10^6^ cells)** | **Extracellular YSBN5**  **(pmol/ 10^6^ cells)** | **Extracellular pHLUM**  **(pmol/ 10^6^ cells)** | **Extracellular SeMeCo**  **(pmol/ 10^6^ cells)** |
| --- | --- | --- | --- | --- |
| Q | 3178.84 ± 159.17 | 52.50 ± 5.93 | 75.23 ± 7.13 | 155.53 ± 17.67 |
| E | 9147.08 ± 410.78 | 48.27 ± 4.47 | 65.20 ± 11.67 | 159.93 ± 2.42 |
| A | 10445.80 ± 771.80 | 23.85 ± 2.79 | 49.41 ± 6.33 | 92.42 ± 34.40 |
| D | 294.68 ± 51.31 | 14.07 ± 1.13 | 22.93 ± 6.64 | 56.08 ± 4.04 |
| R | 3478.80 ± 455.95 | 13.56 ± 0.30 | 23.32 ± 5.52 | 73.39 ± 25.09 |
| S | 160.80 ± 25.44 | 12.60 ± 1.32 | 20.55 ± 6.56 | 33.52 ± 1.34 |
| G | 277.19 ± 3.68 | 11.84 ± 1.04 | 20.14 ± 6.94 | 35.49 ± 2.26 |
| Y | 224.13 ± 16.06 | 7.99 ± 1.04 | 14.23 ± 2.04 | 31.54 ± 6.05 |
| CIT | 336.08 ± 27.35 | 7.02 ± 0.25 | 10.77 ± 3.27 | 21.52 ± 0.91 |
| H | 824.45 ± 74.18 | 6.83 ± 0.75 | 11.33 ± 3.23 | 23.70 ± 1.50 |
| V | 969.59 ± 105.29 | 6.68 ± 0.76 | 10.81 ± 2.31 | 19.31 ± 3.19 |
| O | 157.45 ± 11.53 | 6.09 ± 0.24 | 9.35 ± 2.13 | 21.49 ± 0.87 |
| U | 14.66 ± 1.03 | 3.97 ± 0.41 | 10.80 ± 2.20 | 86.83 ± 16.55 |
| T | 77.85 ± 1.16 | 3.46 ± 0.10 | 6.61 ± 1.37 | 13.30 ± 0.45 |
| N | 281.04 ± 12.60 | 3.75 ± 0.15 | 5.22 ± 1.01 | 11.41 ± 0.78 |
| I | 124.72 ± 3.44 | 3.76 ± 0.37 | 5.57 ± 1.36 | 10.55 ± 0.53 |
| K | 167.25 ± 3.00 | 3.76 ± 0.39 | 6.21 ± 2.40 | 13.86 ± 1.10 |
| P | 294.02 ± 13.38 | 2.88 ± 0.29 | 3.35 ± 0.66 | 8.03 ± 0.57 |
| L | 78.87 ± 5.02 | 2.67 ± 0.42 | 3.82 ± 2.22 | 4.58 ± 2.01 |
| F | 74.11 ± 5.85 | 0.95 ± 0.09 | 1.68 ± 0.74 | 3.40 ± 1.00 |
| M | 9.51 ± 1.47 | 0.39 ± 0.06 | 0.62 ± 0.26 | 1.46 ± 0.09 |
| W | 11.89 ± 0.15 | 0.37 ± 0.06 | 0.50 ± 0.25 | 0.92 ± 0.09 |

**Figure 3- source data 1: Absolute quantification of amino acids and uracil in yeast strains YSBN5, pHLUM and SeMeCo.** All strains grown to exponential growth phase prior to metabolite extraction.
