# Supplementary material for: Self-establishing communities enable cooperative metabolite exchange in a eukaryote
Source: eLife. 2015 Oct 26;4:e09943. doi: 10.7554/eLife.09943 (PMC4695387; doi:10.7554/eLife.09943)
Supplement: Figure 4—source data 1. — DOI: http://dx.doi.org/10.7554/eLife.09943.015 [file elife-09943-fig4-data1.docx]

Figure 4- source data 1

| **Plasmid** | **Marker** | **Fluorescence** | **Segregation rate (%)** | |
| --- | --- | --- | --- | --- |
|  |  |  | **Individual** | **All four** |
| yEpCFP_HIS | *HIS3* | Cyan | 3.87 ± 0.68 | 3.05 ± 0.26 |
| yEpSapphire_LEU | *LEU2* | Sapphire | 4.34 ± 0.85 | 5.01 ± 0.44 |
| yEpVenus_URA | *URA3* | Venus | 3.44 ± 0.37 | 5.39 ± 0.98 |
| pRS411-*GPD*pr-mCherry | *MET15* | Cherry | 2.35 ± 0.39 | 2.07 ± 0.80 |

**Figure 4- source data 1: Segregation rates of fluorescent protein plasmids from the yEp, pRS and p400 series**
